# Books and Pamphlets Received

**Published:** 1884-12-20

**Authors:** 


### BOOKS AND PAMPHLETS RECEIVED.

*Medical Visiting List*, or Physicians' Diary for 1885. New York: Wm. Wood & Co., an admirably arranged visiting list, with special memoranda for Physicians.

"Wm. Wood & Co. issued this little book, as they do all their publications; excellence and cheapness are marvelously combined by them, and repeatedly evoke praise from the impartial and elicit wonder from their competitors."

*Physicians' Pocket Day Book*, designed by C. Henri Leonard, M. A., M. D., Prof. of Medical and Surgical Diseases of Women and Clinical Gynecology in the Michigan Medical College, Member of the American Medical Association, etc. Accommodates daily charges for twenty-five or fifty families. Has complete Obstetrical Record for ninety-four cases and monthly memoranda for Dr. and Cr. cash account.

Price, $1.00; your name aud year on the side in gold leaf, $1.25; name, town, state and year, $1.50. Issued annually by Illustrated Medical Journal Co., Detroit, Mich.

*Transactions of the Michigan State Medical Society for the year* 1884.

The meeting of the Society was at Grand Rapids, June 11th and 12th last. It is a creditable book, and contains quite a number of able and interesting papers. The following are the officers elect for 1884:

President—Donald McLean, M.D., Detroit, Mich.
First Vice-President—J. Perkins, M.D., Owosso, Mich.
Second Vice-President—J. M. Cook, M.D., Muskegon, Miss.
Third Vice-President—Gordon Chettock, M.D., Jackson, Miss.
Fourth Vice-President—Carl Brumem, M.D., Detroit, Mich.
Secretary—Geo. E. Rauney, M.D., Lansing, Mich.
Treasurer—A. R. Smart, M.D., Hudson, Mich.
The next meeting will be at Port Huron, on Wednesday, June 10th, 1885.

*Reports of the Trustees and Officers of the Lunatic Asylum of the State of Georgia* from October 1st, 1883, to October 1st, 1884.

We are indebted to the kindness of the able Superintendent of the Asylum, Dr. T. O. Powell, for a copy of the above Report.

It appears that there were in the Asylum at the time of the Report—

| | |
|---|---|
| White male lunatics | 337 |
| White female lunatics | 383 |
| White male epileptics | 53 |
| White Female epileptics | 45 |
| Male Idiots | 32 |
| Female Idiots | 41 |
| Total | 891 |
| Of the colored inmates there are | 339 |
| In all | 1220 |

The Report of the Trustees makes a fine exhibit as to the sanitary condition and management of the 'Institution. The percentage of mortality compares favorably with other Institutions of the kind, and Dr. Powell, the Superintendent, is commended for his faithfulness and ability, and for his peculiar fitness for the position assigned him. The Board, very properly, we think, recommend an increase of his salary.

*Transactions of the Texas State Medical Association*, held at Belton, April 22, 25, 1884.

The officers of this Association are the following:

President—Dr. H. C. Ghent, of Belton.

Vice-Presidents—Dr. E. P. Becton, Sulphur Springs.
Dr. H. H. Dare, Caldwell.
Dr. M. Matkin, Hearne.

Secretary—Dr. W. J. Burt, Austin.

Treasurer—Dr. J. L. Houston, Houston.

The work contains 246 large octavo pages, and many able and valuable papers.

The next meeting will be at Houston, Texas, 3d Tuesday in April, 1885.

*Creator and Cosmos*, Third Revision, with enlargement, by Robert Shaw, M. A.,

This is a work of the highest order; one that treats of the most profound subjects in religion and science: the Deity; the World in its relation to the Creator, in its physical and moral aspect; the Stars; of all the forces that go to make up the grandeur of the Universe; an examination into the origin of Christianity, with a learned exposition of the Gospels and the Acts—by a man who has devoted his life to the study of these grand subjects—written in a style that brings its points home comprehensively to all.

This valuable and interesting work—an Encyclopædia of Religion and Science—of 2,229 pages, is handsomely printed and bound, and will be sold, by subscription only, at the following rates: Bound in Fine English Cloth, the two volumes in one, $5.00; or, the vols. separate, $3.00 each; bound in leather, library style, the two vols. in one, $6.00; or, the vols. separate, $3.50, each.

*Physicians' Visiting List*, by Lindsay & Blakiston, for 1885. Thirty-fourth year of its publication. Philadelphia: P. Blakiston, Son & Co., successors to Lindsay & Blakiston, 1012 Walnut St., Philadelphia. Sold by all Booksellers & Druggists.

This book contains list and doses of new remedies and other useful information for the practitioner. It has been long in use by the Profession and is deservedly very popular as a visiting list.

*Genital Reflexes*, the result of an Abnormal Physical Condition of the Genital Organs, known as Phimosis, by T. Griswold Comstock, M. A., M. D., St. Louis, Mo.

